# A Randomized Controlled Study on the Effects of a Documentary on Students’ Empathy and Attitudes towards Older Adults

**DOI:** 10.17140/PCSOJ-3-127

**Published:** 2017-07-20

**Authors:** Luciana Laganá, Larisa Gavrilova, Delwin B. Carter, Andrew T. Ainsworth

**Affiliations:** 1 Psychology Department, California State University Northridge, Northridge, CA 91330, USA; 2 Psychological Sciences, University of California, Merced, School of Social Sciences and Humanities, Merced, CA 95340, USA; 3 Quantitative Methods in the Social Sciences, University of California, Santa Barbara, Department of Education, Santa Barbara, CA 93106, USA

**Keywords:** Ageism, Empathy, Older adults, Pain, Randomized controlled trial, Randomized controlled trial (RCT)

## Abstract

**Background:**

Despite the rapid increase in the size of the geriatric population, no current published literature is available based on the effects of viewing a documentary covering medical and psychosocial issues concerning older adults influencing young people’s empathy and ageism. The aim of the current study was to test whether participants who viewed an original documentary about older adults experiencing physical pain would report lower ageism and higher empathy scores when compared to participants who watched a neutral documentary.

**Method:**

Seventy-seven students (ages 18–29 years) were randomized to either the experimental (pain documentary) or the control (neutral documentary) conditions and given pre- and post-test measures of empathy and ageism.

**Results:**

The results of a series of Profile Analyses (Multivariate Mixed ANOVAs) showed a significant interaction (Wilk’s λ=0.933, *F*(1,75)=5.389, *p*=0.023, partial η^2^=0.067) between treatment and time (pre- *vs*. post-viewing the film) for the empathy measure that was confirmed by follow-up *t*-tests. The latter showed a significant increase in empathy scores for only the experimental group, *t*(37)=−2.999, *p*=0.005. However, contrary to the original prediction, this same treatment by time effect was not observed for ageism (Wilk’s λ=0.994, *F*(1,75)=0.482, *p*=0.490, partial η^2^=0.006), as the experimental participants did not significantly reduce their ageism scores, *t*(38)=0.725, *p*=0.473. The results of these analyses, as well as those obtained by using the subscales of each questionnaire, have been discussed.

**Conclusions:**

The findings of this preliminary study indicate that showing a pain-based, anti-bias documentary feature film has the potential to significantly improve empathy towards older adults in university students.

## INTRODUCTION

With populations aging rapidly, ageism is becoming a pronounced social issue that will affect societies throughout the world in the coming decades. Although aging as an individual is an inevitable biological process for every living person, our society holds negative stereotypes and prejudices towards older adults in the form of ageism.^1–2^ According to Butler, ageism is “a process of systematic stereotyping and discrimination against people because they are old”.^3^ Even though prejudice and discrimination come in many forms, ageism has not been given as much research attention as it deserves. Compared to sexism or racism, for example, research on negative attitudes towards older populations is scant.^4–5^ Yet, ageism may be the most commonly held form of negative stereotype and the most experienced form of discrimination.^6^

The growing profusion of age-related stereotypes and discrimination is driven, at least in part, by various media outlets (e.g., television, movies, magazines, and social media). While media has the potential to positively influence people to be more understanding and less discriminatory towards older adults, the opposite often occurs. As society places greater importance on physical appearance, beauty and youth can easily be idolized, while aging is typically associated with physical and cognitive impairments and dependence.^7–8^ In this regard, higher levels of internalization of North American appearance ideals among undergraduate students have been associated with an expression of more negative attitudes towards older adults.^9^ A striking similarity exists between ageism and the discrimination of disabled people, as older adults are often perceived as physically incapable and sick.^10–11^ Additionally, the media sends frequent messages to young individuals that older people are an economic burden to society,^12^ further augmenting the divide between younger and older adults.

The aforementioned age-related divide has also been linked to lowered feelings of empathy towards older adults in society.^13^ Empathy has been classically defined as an emotional response to someone else’s situation or emotional state.^14^ When feeling empathy, an individual experiences someone else’s affective responses.^15^ Ageism is a form of prejudice that many people hold towards their own potential future selves^5,16^ as a method to reduce their anxiety related to aging and death; thus, it often leads to individuals categorizing older adults under an “other” group or out-group in which the younger individual does not belong.^17^ Placing older adults into an out-group helps people a) distance themselves from older adults and b) externalize the impact of aging. This distancing process can be linked to the individuals’ efforts to diminish the anxiety that they feel towards their own future^1,5^ and, consequently, could diminish interpersonal empathy towards older adults as well as increase ageist attitudes.

The spread of ageist views and discriminatory behavior against older adults has the potential to impact everyone in the society, even the very professionals who are supposed to care for older adults. Researchers have highlighted that physicians and nurses often hold negative attitudes towards older patients^18^ and are less empathic towards them compared to younger patients. There are several consequences of holding negative attitudes towards older adults that could have deleterious effects on the lives of older people. For instance, ageism among health professionals posits notable danger because it may impact the quality of care provided to older patients.^19–20^ In fact, healthcare professionals agree that older adults receive a lower quality of care compared to younger patient populations.^21^ Many physicians and nurses perceive older adults as weak, demented, ill, and intolerant,^18^ and they often report that they do not like working with older individuals.^22–23^ Consequently, efforts to reduce ageism among healthcare professionals could have a major positive impact on the quality of life of older adults, as they could contribute to giving aging individuals access to a better quality of healthcare.^19–20^

Research related to combatting ageist beliefs has shown that gaining education related to ageing is associated with increased empathy and enhanced attitudes towards older adults.^24–26^ This is the case especially among students in the helping professions,^25,27^ but research studies on the link between attitudes towards older adults and intervention strategies are limited.^28^ In particular, experiential learning (e.g., service learning, internship, and field placement) can lead to lower ageism.^29^ In this regard, medical students who completed a geriatric clerkship as part of their internal medicine rotation reported a more positive attitude towards older adults.^30^ Furthermore, in two investigations on this topic, Kumagai^31,32^ used a story-telling approach to improve attitudes among medical students as students paid home visits to the family of a patient affected with chronic conditions during the two years of their program. After a scheduled home visit and listening to the volunteers’ life stories, students participated in small group discussions with their instructor in the first study; in the follow-up study on the effects of “diabetes stories”, students were asked to reflect on how their understanding of chronic illness differed from knowledge gained through lectures and textbooks. Qualitative analyses of both studies showed that this intervention promoted a better understanding of chronic illness and its management.

Getting individuals to understand, connect with, and and feel empathy towards older adults could be the key to reducing ageism. Regarding having empathy towards older adults, researchers have documented that perspective-taking and empathy are mediators of the relationship between intergroup contacts (like those between young adults and older adults) and prejudice.^33^ Others have noted a relationship between empathy and reduced ageist beliefs.^13^ Furthermore, empathy has been shown to be impacted by media exposure. For example, emotional responses to film characters have been studied by Kincaid,^34^ who postulated that an empathic emotional response to film viewing is a motivational force that could make viewers reconceptualize the central problems depicted in a film. In line with this conceptualization, researchers^35^ showed video clips of encounters with medical patients (not specifically geriatric patients) to medical students assigned to the experimental condition. This viewing led to higher empathy scores compared to the scores obtained by the control participants, who watched a neutral film.

The present study was intended to shed light on the linkages between a documentary film-based intervention, empathy, and ageism towards older adults. Currently, no published study is available on the anti-bias effects of a documentary-based intervention targeting older adults’ pain, suffering, and resilience. Building on the work of Hojat et al^35^ and of Kumagai et al^31–32^, the current study was conducted with the intent to investigate whether a documentary-style, anti-ageism film that highlights the resilience of older adults and their ability to function despite untreated or under-treated physical pain could lead to experiencing a positive reconceptualization of older adults among young viewers. Utilizing a film-based intervention instead of in-person training or experiential learning has the added advantage of reaching a wider audience with minimal effort or funding compared to in-person training. Furthermore, a media-based intervention has the potential to act as a surrogate or proxy for a one-on-one, in-person experience with older individuals, which has been shown to increase empathy and reduce ageism towards older adults.

In this pilot randomized controlled trial (RCT), in line with the previously summarized literature, the authors expected that viewing an original documentary film featuring resilient seniors living with physical pain, as well as experts explaining the difficulties associated with treating physical pain in older age, would shed light on this topic and possibly improve attitudes and empathy towards older adults. It was hypothesized that participants who viewed this film would report significantly lower ageism and higher empathy scores when compared to participants assigned to the control condition (i.e., the viewing of a neutral documentary of the same duration).

## METHOD

### Participants

Research participants were 77 undergraduate students aged from 18–29 years, from a diverse state university, who volunteered for the study to obtain course credit; 38 participants were assigned to the experimental group and 39 participants were assigned to the control group. The study was open to any undergraduate student enrolled in the research participant pool, with the exception of students who had been enrolled in courses related to the study of older adults (e.g., gerontology), in order to reduce the chances of recruiting students who were particularly well-disposed towards older adult populations. The sample was composed predominantly of women (*n*_women_=57 and *n*_men_=20) and was ethnically diverse, reflecting the composition of the psychology department and the vast ethnic diversity of the university’s student population; aggregate and group demographic information is contained in Table 1.

### Materials

#### Questionnaires

The authors utilized validated scales that measure both empathy and ageism as well as a questionnaire covering demographic information about the participants. The demographics questionnaire has been utilized by the first author in several prior research projects and asks questions pertaining to age, gender, ethnicity, education, marital status, employment, and religion. Empathy was quantified using the Jefferson Scale of Empathy (JSE) Health Professions Student (HPS) version. The version of the JSE-HPS utilized in the present study is a modified 17-item version that was designed to measure empathy in university students within healthcare fields (see Williams, Brown, Boyle, & Dousek^36^ for details on the modification). The JSE-HPS items are measured on a Likert scale, with response choices ranging from 1 “Strongly Disagree” to 7 “Strongly Agree”. Williams and colleagues^36^ identified two subscales within the JSE-HPS: Compassionate Care and Perspective Taking. The Compassionate Care subscale contains 9 items, with statements like “I believe that emotion has no place in the treatment of medical illness” (reverse coded). The Perspective Taking sub-scale contains 8 items, with statements such as “Healthcare providers should try to stand in their patients’ shoes when providing care to them”. The JSE-HPS total showed acceptable reliability in the current sample (Cronbach’s α=0.803) as did the Perspective Taking subscale (Cronbach’s α=0.809). However, the Compassionate Care subscale had a slightly sub-optimal reliability (Cronbach’s α=0.751). Ageism was measured using North and Fiske’s Succession, Identity and Consumption (SIC) measure of ageism.^37^ The SIC is a 20-item scale designed to quantify intergenerational-tension; it was developed and validated on both university and non-university participants. Its items are measured on a Likert scale, and choices range from 1 “Strongly Disagree” to 6 “Strongly Agree”. This measure was designed to capture the three subscales identified in the title of the scale: Succession, Identity and Consumption. The Succession subscale contains 8 items, with statements like “Most older people don’t know when to make way for younger people”. The Identity sub-scale contains 5 items, with statements such as “Older people typically should not go to places where younger people hang out”. Finally, the Consumption subscale contains 7 items, with statements like “Doctors spend too much time treating sickly older people”. The SIC total and all three subscales showed acceptable levels of reliability in the current sample (SIC Total: Cronbach’s α=0.886; Succession: Cronbach’s α=0.842; Identity: Cronbach’s α=0.847; Consumption: Cronbach’s α=0.821).

#### Documentary films

This study’s first and the second authors created a 90-minute documentary titled “Understanding Pain in Older Age”, which highlights the challenging nature of treating pain in older adults. The film features interviews with older individuals who share their personal stories about a) living with chronic pain due to health conditions, and in some cases b) being unable to take enough or any pain medication to successfully manage their pain, for a variety of reasons such as liver problems. Additionally, it covers interviews with health professionals, including pain experts who discuss challenges of managing physical pain in older age. This documentary has been featured at independent film festivals and has garnered recognition for its social impact efforts regarding pain and the aging adult population. The documentary film for the neutral condition was a nature documentary titled “Kiwi Country New Zealand”; it features several natural locations of New Zealand. This documentary was chosen because 1) when additional footage on New Zealand parks (available as bonus footage on the DVD) was added to it, the film had the same pain documentary, 2) it won similar awards at independent film festivals, and 3) its content is thematically neutral relative to older adults and ageism.

### Procedures

This investigation was approved by the Institutional Review Board governing research conducted by the first author. The study’s introduction, experiment, and debriefing were conducted in a large sized classroom that was equipped with a film projector, tables and chairs. In order to test whether watching a pain documentary could impact ageism and empathy, the authors created two experimental groups by randomizing participants (*via* tossing a coin) into either the experimental (i.e., pain documentary viewing) or control group (i.e., neutral documentary viewing), thus creating two randomly equivalent groups prior to treatment (see Table 1 for a list of demographic information by group). Small groups of experimental and control participants participated at separate times; the pre- and post-test data collection as well as film viewing all occurred in the same room for all participants. After a general introduction and informed consent process, students were administered a pre-test consisting of the three questionnaires (i.e., demographics, empathy, and ageism) described above. Each group of students then proceeded to watch their respective 90-minute documentary film; afterwards, participants were asked to complete a post-test survey that contained the same ageism and empathy questionnaires filled out in the pre-test. Once the post-test was completed, participants were debriefed and the study was concluded. As this was a pilot study to investigate the possible use of a documentary film as an intervention method, participants were not probed for suspicion or demand characteristics following treatment/exposure to the film.

### Analytic Strategy

Prior to performing any inferential analyses on the study’s variables, the analytical plan included calculating descriptive statistics (i.e., means, standard deviations, and frequencies) for both the Empathy and the Ageism scales as well as for the demographic items. Next, the authors planned to conduct Profile Analyses (Multivariate Mixed ANOVAs) to verify that an interaction effect was present, namely, whether scores for Ageism and Empathy moved significantly and in the correct direction for the experimental but not the control group. Subsequent to the multivariate analysis, the analytical strategy included conducting paired samples *t*-tests to further compare pre- and post-ageism and empathy scores separately in each group (control *vs*. treatment).

## RESULTS

The collected data was analyzed using the SPSS-PC software version 22. All statistical tests employed an alpha level of 0.05. Profile analyses (i.e., multivariate mixed ANOVAs) were carried out for the total and subscale scores for both Empathy and Ageism measures with Time (Pre *vs*. Post) as the within-subjects factor and Group (treatment *vs*. control) as the between-subjects factor. Profile analyses provide tests for aggregate change over time (i.e., ignoring group membership), group differences^(footnote1)^ (i.e., collapsing the pre- and post-test scores), and a test for the interaction between Time and Group. Prior to conducting the analyses, no univariate or multivariate outliers were identified in the dataset (*α*=0.001).^38^ Additionally, data screening revealed that the assumptions of normality, homogeneity of variance-covariance matrices, linearity, and multicollinearity were met, thus the dataset was suitable for the planned analyses.

### Empathy

**Figure 1:**
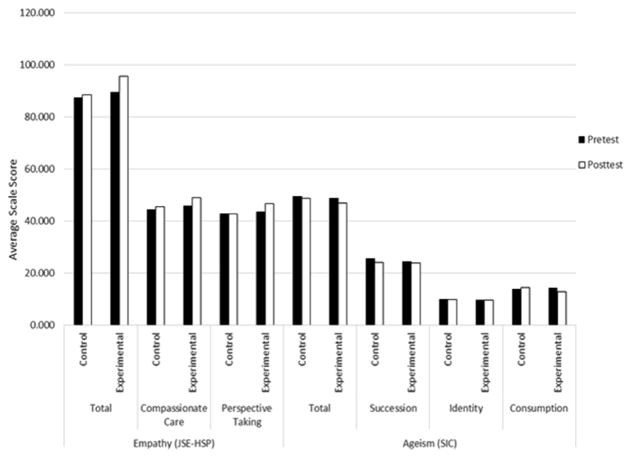
Average Pre- and Post-Test Total and Subscale Scores for the Empathy and Ageism Measures for the Control and Experimental Groups.

A profile analysis was performed on the basis of the total JSE-HPS scores; the findings indicated that the univariate effect for the Group was not significant *F*(1,75)=3.161, *p*=0.079, partial η^2^=0.040). Results for the multivariate tests indicated a significant difference for the main effect of Time (Wilk’s λ=0.877, *F*(1,75)=10.557, *p*=0.002, partial η^2^=0.123). The 2-way interaction between Time and Group was also significant (Wilk’s λ=0.933, *F*(1,75)=5.389, *p*=0.023, partial η^2^=0.067; see Figure 1). Analyses of each of the subscales indicated that, for Compassionate Care, the effect for Time was significant (Wilk’s λ=0.924, *F*(1,75)=6.186, *p*=0.015, partial η^2^=0.076), but the effects of both Group (*F*(1,75)=2.266, *p*=0.136, partial η^2^=0.029) and the interaction (Wilk’s λ=0.981, *F*(1,75)=1.474, *p*=0.228, partial η^2^=0.019) were not significant. Regarding the Perspective Taking subscale, the interaction was significant (Wilk’s λ=0.950, *F*(1,75)=3.964, *p*=0.050, partial η^2^=0.050). However, the effects of Group (*F*(1,75)=2.142, *p*=0.147, η^2^=0.028) and Time (Wilk’s λ=0.953, *F*(1,75)=3.704, *p*=0.058, partial η^2^=0.047) were not significant, as illustrated in Figure 1.

### Ageism

A profile analysis was performed on the basis of the total SIC scores; the results indicated that the univariate effect for Group was not significant *F*(1,75)=0.128, *p*=0.721, η^2^=0.002). Results for the multivariate tests indicated no significant difference for the main effect of Time (Wilk’s λ=0.968, *F*(1,75)=2.472, *p*=0.120, partial η^2^=0.032). The 2-way interaction between Time and Group was not significant (Wilk’s λ=0.994, *F*(1,75)=0.482, *p*=0.490, partial η^2^=0.006; see Figure 1). Analyses of each of the subscales resulted in the following findings: 1) Succession: the Time effect was significant (Wilk’s λ=0.950, *F*(1,75)=3.966, *p*=.050, partial η^2^=0.050); however, the Group (*F*(1,75)=0.007, *p*=0.782, η^2^=0.001) and interaction (Wilk’s λ=0.990, *F*(1,75)=0.728, *p*=0.396, partial η^2^=0.010) effects were not significant; 2) Identity: Group (*F*(1,75)=0.022, *p*=.883, partial η^2^=0.0002), Time (Wilk’s λ=1.000, *F*(1,75)=0.003, *p*=0.955, partial η^2^=0.00004), and interaction (Wilk’s λ=1.000, *F*(1,75)=0.032, *p*=0.859, partial η^2^=0.0004) effects were not significant; and 3) Consumption: Group (*F*(1,75)=0.188, *p*=0.666, partial η^2^=0.002), Time (Wilk’s λ=0.994, *F*(1,75)=.425, *p*=0.517, partial η^2^=0.042), and interaction (Wilk’s λ=0.958, *F*(1,75)=3.253, *p*=0.075, partial η^2^=0.042) effects were not significant (see Figure 1).

### Paired Sample *t*-test Results

Table 2 illustrates the descriptive statistics for the total and sub-scale scores for Empathy and Ageism, as well as the correlational values and the results of the paired samples *t*-tests (i.e., post-test minus pre-test score). Results of the paired sample *t*-tests on the total scores indicated that there was a significant mean difference in Empathy scores for the experimental group (*t*(37)=2.999, *p*=0.005), but not for the control group (*t*(38)=1.175, *p*=0.247). However, there were no significant mean differences for Ageism scores for either the experimental (*t*(37)=−1.417, *p=*0.165) or control (*t*(38)= −0.725, *p*=0.473) group. The significant difference between the pre- and post-test scores on Empathy for the experimental group was primarily driven by a significant change in the post-test scores on the Perspective Taking subscale (*t*(37)=2.191, *p*=0.035). The change on the Compassionate Care subscale for the experimental group was right on the cusp of significance as well (*t*(37)=2.011, *p*=0.052). Table 2 does indicate an effect for the Succession subscale of the Ageism measure in that, although the overall scale did not show a significant change for either group, there was a significant decrease for the control group on this one subscale (*t*(38)= −2.299, *p*=0.027). Additionally, Table 2 shows that the correlations between pre- and post-test scores on both the Empathy and Ageism scales were very high (indicating that participants responded consistently across time) for the control group participants, while for the experimental group the correlations were often considerably lower compared to the control group. For example, for the overall measure of Empathy, in the experimental group the correlation was relatively low (*r*=0.468) when compared to the correlation on the same measure in the control group (*r*=0.914).

## DISCUSSION

In the present experiment, the authors examined whether viewing a pain-focused documentary could affect the perception of older adults and their many medical struggles among college students, with the intent of improving empathy and attitude towards them. Similar to previous research on the impact of exposure on empathy,^13,24–27,30–32,35^ the significant between Group and Time found in the Profile Analysis results indicated that viewing the pain documentary did in fact impact experimental participants’ empathy scores positively and significantly. Follow-up *t*-test analyses were performed in order to delineate this effect; it was discovered that the impact of viewing the pain documentary was reflected in the significant changes in participants’ empathy scores, and this effect was only found in the experimental group. Further investigation into the sub-scales of the empathy measure revealed that what was driving the interaction for the overall empathy score was a significant change in the perspective taking subscale for the experimental group. No significant improvements in total ageism scores were recorded in either group; however, there was a significant, albeit somewhat unexplainable, improvement on the Succession sub-scale of the SIC for the control group. A potential explanation is that, perhaps, given that some portions of the control film were narrated by an older adult, this could have led to the result in question. In view of the effect size values for many of the non-significant effects, it is speculated that conducting this study on larger sample, a process already underway, could lead to achieving additional significant improvements on the Compassionate Care subscale of the JSE-HPS as well as on the Consumption subscale of the SIC.

This study has some valuable implications for designing interventions to improve empathy and attitude towards older adults. Successful interventions may help reduce inter-generational tension, improve quality of life of older adults, and eventually provide them with better access to healthcare, work resources, and possibly even political representation. These interventions could also help decrease a growing problem of recruiting and retaining healthcare providers to geriatric professions.^39^ Furthermore, conducting successful interventions in this area may help our society become better prepared to meet the demands of a multigenerational society characterized by an increasingly growing geriatric population.

### Limitations

This study had its own limitations. First of all, the sample size was modest (n=77), especially in view of the relatively low correlation for pre- and post-empathy scores in the experimental group, which indicated that the effect that the documentary had might have depended on additional participant information not assessed in the study. This relatively low correlation suggests that, while experimental participants increased their empathy on average after viewing the pain-focused film, their scores did not remain as consistent as they did regarding the Empathy measure in the control group (or even for the ageism measure in the experimental group). In other words, some participants’ empathy did increase relative to their starting position but, for others, empathy either did not change or changed in the opposite direction, which is why the correlation was somewhat low. To investigate the above-mentioned issue further, a larger sample is needed in order to include additional variables in the analyses such as age (even within a university student population, as the current study had a relatively wide age range of 18–29 years), gender, as well as regular interaction with older adults as potential moderators of the effect of the intervention on the outcome variables.

Another limitation, common to many studies of this kind, is the fact that the post-test occurred immediately after film viewing. First, having achieved a significant change in empathy scores in a short time is a remarkable accomplishment of the intervention, although it does not allow to ascertain whether these changes will remain after the participants leave the study, nor does it allow for the investigation of whether the empathy changes found immediately in this study will lead to changes in ageism later. Secondly, utilizing a pre-test and post-test in a rather quick succession makes it difficult to confirm whether the changes were in fact real. For instance, using a pre-test and post-test of the same measures in a short amount of time may pose a risk of participants remembering their answers to the pre-test (e.g., urging them not to change their answers) or guessing a what answers researchers may expect from participants – this is a shortcoming of randomized controlled research of this kind in general. In a similar way, there could be an issue of demand characteristics, as participants who are given pre- and post-tests that relate to the intervention may alter their response behavior to affirm what the researchers are investigating. However, the fact that this study’s participants changed on empathy and not on ageism after viewing a documentary featuring older adults makes this concern less critical.

Moreover, considering that two of this article’s authors are the creators of the experimental film, this could create inherent bias. This bias could stem from the fact that some participants might have recognized the film as being made by the team of a CSUN professor and could manifest, for instance, in the authors’ interpretation of outcomes and in how their research team interacted with the participants. Additionally, the study is limited in its generalizability because the demographic makeup of the current sample is not necessarily comparable to other samples of university students. For instance, women and Hispanic-Latino/a students were naturally oversampled in the current study, as this reflects the gender and ethnic composition of the undergraduate student population utilized in the study. Furthermore, the present findings cannot be easily generalized to students from other dissimilar educational institutions nor to non-students of the same age group in general.

### Future Directions

As a pilot study with promising results, the current investigation is the first step of many towards truly understanding the impact that a documentary film can have on young individuals’ attitudes towards older adults. To begin with, future research in this area should ideally be conducted on a larger scale in order to include and parse out additional variables that could be impacting the effect of the intervention on potential outcomes. Interested researchers should not only investigate the potential moderating effects of demographic group membership (e.g., age and gender) to further elucidate the cause of the inconsistent responding from pre- to post-test on some of the measures for the experimental group, but also multiple factors that are potentially related to the outcome variables. As an example of additional factors, some evidence suggests that death anxiety and ageism are highly correlated.^40–42^ Death anxiety can make young people distance themselves from older adults as a coping mechanism through which to avoid confronting their own future selves and the inevitability of their own death.^16^ Relating this information to the current study, it is possible that, for respondents in the treatment group who had a high death anxiety level, viewing the pain film might have triggered this anxiety and led to a decrease in empathy towards older adults. Another example of a group of variables that could be included in future studies using larger sample sizes is the impact of mass media (to be measured covering several factors) on the two outcome variables in question. For instance, as reported earlier, research indicates that young adults are bombarded with mass media messages that lead them to internalize 1) youthful ideals, with beauty and youth being idolized and becoming older being related to physical and cognitive impairments as well as dependence,^7–8^ and 2) ageist messages, as young people are told that older adults are economic burdens to society.^12^ Future studies could cover the extent of research participants’ internalization of these types of messages in order to explore their mediating or moderating impact on the effects of interventions like the one tested herein.

Moreover, although a randomized controlled trial design was used in the current study, researchers could consider implementing a variety of methodological improvements (e.g., manipulation checks, probes for demand characteristics during debriefing, varying times between pre- and post-tests, implementing statistical controls, utilizing more sensitive outcomes measures) in order to ensure significant findings that are meaningful. For instance, interested scholars could actively attempt to reduce participant-related artifacts that could impact the study (e.g., demand characteristics) and include questions that would 1) verify whether the intervention was fully recognized by the participant (manipulation check), 2) investigate whether the participant was suspicious of minor deceptions used to mask the point of the study, and/or 3) assess participant’s demand characteristics (e.g., “good-participant” effect) in order to fully address them during the analysis phase. In view of the limitations of the pre-test and post-tests timing mentioned, researchers could purposely vary the length of time between the pre- and post-tests in order to increase the generalizability of the effect and limit the impact of phenomena like carry-over effects. Furthermore, in the current study, it was not possible to parcel out the effects of viewing the painful film on specific aspects of empathy related to the intervention (e.g., empathy towards pain *vs*. towards older individuals *vs*. towards older adults in pain), another common shortcoming of studies of this kind. Future research could also include the utilization of outcome measures that are sensitive enough or broad enough in scope for a more nuanced investigation into how participants’ empathy and ageism were impacted by the intervention.

### Conclusive Comments

This preliminary study, which could be viewed as an anti-bias educational exercise, is a promising first step towards testing whether film-based interventions can increase empathy and decrease age-related bias against older adults.

## Figures and Tables

**Table 1:** Demographic Characteristics of the Combined Sample (N=77) and of Each Group (Experimental n=38, Control n=39).

Demographic Variable	Overall		Experimental		Control	

	N	Statistic	N	Statistic	N	Statistic
**Age (Range is 18–29years)**						
Mean		19.403		19.158		19.641
Standard Deviation		1.873		1.128		2.378

**Gender**						
Women	57	74.0%	30	78.9%	27	69.2%
Men	20	26.0%	8	21.1%	12	30.8%

**Classification**						
Freshman	39	50.6%	21	55.3%	18	46.2%
Sophomore	24	31.2%	13	34.2%	11	28.2%
Junior	9	11.7%	3	7.9%	6	15.4%
Senior	5	6.5%	1	2.6%	4	10.3%

**Race/Ethnic Background**						
White/Caucasian	10	13.0%	5	13.2%	5	12.8%
White/Mixed	2	2.6%	2	5.3%	0	0.0%
Black/African American	3	3.9%	2	5.3%	1	2.6%
Black/African American Mixed	1	1.3%	1	2.6%	0	0.0%
Hispanic/Latino	48	62.3%	22	57.9%	26	66.7%
Asian/Pacific Islander	9	11.7%	4	10.5%	5	12.8%
Other	4	5.2%	2	5.3%	2	5.1%

**Marital Status**						
Single	72	93.5%	36	94.7%	36	92.3%
Married	2	2.6%	0	0.0%	2	5.1%
Living with Significant Other	3	3.9%	2	5.3%	1	2.6%

**Employment Status**						
Not Employed	47	61.0%	21	55.3%	26	66.7%
Part-time	25	32.5%	15	39.5%	10	25.6%
Full-time	5	6.5%	2	5.3%	3	7.7%

**Total Income**						
Less than $20,000	21	27.3%	11	28.9%	10	25.6%
$20,000 – $39,000	29	37.7%	13	34.2%	16	41.0%
$40,000 – $59,000	7	9.1%	4	10.5%	3	7.7%
$60,000 – $79,000	6	7.8%	4	10.5%	2	5.1%
$80,000 – $99,000	5	6.5%	1	2.6%	4	10.3%
$100,000 and above	9	11.7%	5	13.2%	4	10.3%

**Religion that influences you**						
Christianity	51	66.2%	27	71.1%	24	61.5%
Judaism	1	1.3%	0	0.0%	1	2.6%
Islam	3	3.9%	3	7.9%	0	0.0%
Hinduism	1	1.3%	0	0.0%	1	2.6%
Other	21	27.3%	8	21.1%	13	33.3%

**Table 2:** Descriptive Statistics and *t*-Test Results for the Total and Subscales Scores of the Empathy and Ageism Scales.

			Pre-test		Post-test			Pearson	Post-, Pre- Mean Difference				
	Scale	Group	M	SD	M	SD	n	r	95% CI		*t*-value	df	sig.
**Empathy (JSE-HSP)**	Total	Control	87.436	12.219	88.462	13.432	39	0.914*	−0.741	2.793	1.175	38	0.247
		Experimental	89.500	13.838	95.658	9.721	38	0.468*	1.998	10.318	2.999	37	0.005*
	Compassionate	Control	44.513	7.196	45.590	8.840	39	0.872*	−0.337	2.491	1.542	38	0.131
	Care	Experimental	45.921	9.652	49.053	5.789	38	0.309	−0.024	6.287	2.011	37	0.052
	Perspective	Control	42.923	6.823	42.872	7.230	39	0.797*	−1.507	1.404	−0.071	38	0.944
	Taking	Experimental	43.579	8.617	46.605	6.804	38	0.410*	0.227	5.825	2.191	37	0.035*
	Total	Control	49.487	14.133	48.692	16.579	39	0.913*	−3.015	1.425	−0.725	38	0.473
		Experimental	48.895	16.523	46.842	14.487	38	0.842*	−4.989	0.883	−1.417	37	0.165
**Ageism (SIC)**	Succession	Control	25.590	8.861	24.077	10.017	39	0.912*	−2.845	−0.181	−2.299	38	0.027*
		Experimental	24.605	7.557	24.000	8.217	38	0.788*	−2.306	1.096	−0.721	37	0.475
	Identity	Control	9.974	4.960	10.026	5.096	39	0.955*	−0.442	0.544	0.211	38	0.834
		Experimental	9.842	5.320	9.816	5.281	38	0.910*	−0.765	0.713	−0.072	37	0.943
	Consumption	Control	13.923	4.636	14.590	6.021	39	0.812*	−0.474	1.808	1.183	38	0.244
		Experimental	14.447	7.199	13.026	5.206	38	0.525*	−3.488	0.646	−1.393	37	0.172

CI=Confidence interval,

* *p*<0.05.

## Footnotes

1 This is a univariate test because each participant’s score used in this test is an average across the within-subjects factors.
